# Calcium Channels and Oxidative Stress Mediate a Synergistic Disruption of Tight Junctions by Ethanol and Acetaldehyde in Caco-2 Cell Monolayers

**DOI:** 10.1038/srep38899

**Published:** 2016-12-13

**Authors:** Geetha Samak, Ruchika Gangwar, Avtar S. Meena, Roshan G. Rao, Pradeep K. Shukla, Bhargavi Manda, Damodaran Narayanan, Jonathan H. Jaggar, RadhaKrishna Rao

**Affiliations:** 1 Department of Physiology, University of Tennessee Health Science Center, 894 Union Avenue, Memphis TN 38163, USA.

## Abstract

Ethanol is metabolized into acetaldehyde in most tissues. In this study, we investigated the synergistic effect of ethanol and acetaldehyde on the tight junction integrity in Caco-2 cell monolayers. Expression of alcohol dehydrogenase sensitized Caco-2 cells to ethanol-induced tight junction disruption and barrier dysfunction, whereas aldehyde dehydrogenase attenuated acetaldehyde-induced tight junction disruption. Ethanol up to 150 mM did not affect tight junction integrity or barrier function, but it dose-dependently increased acetaldehyde-mediated tight junction disruption and barrier dysfunction. Src kinase and MLCK inhibitors blocked this synergistic effect of ethanol and acetaldehyde on tight junction. Ethanol and acetaldehyde caused a rapid and synergistic elevation of intracellular calcium. Calcium depletion by BAPTA or Ca^2+^-free medium blocked ethanol and acetaldehyde-induced barrier dysfunction and tight junction disruption. Diltiazem and selective knockdown of TRPV6 or Ca_V_1.3 channels, by shRNA blocked ethanol and acetaldehyde-induced tight junction disruption and barrier dysfunction. Ethanol and acetaldehyde induced a rapid and synergistic increase in reactive oxygen species by a calcium-dependent mechanism. N-acetyl-L-cysteine and cyclosporine A, blocked ethanol and acetaldehyde-induced barrier dysfunction and tight junction disruption. These results demonstrate that ethanol and acetaldehyde synergistically disrupt tight junctions by a mechanism involving calcium, oxidative stress, Src kinase and MLCK.

A significant body of evidence indicates that alcohol consumption causes gut barrier dysfunction leading to increased endotoxin flux from the intestinal lumen into the mucosa, and eventually into the blood circulation causing the condition, “Alcoholic Endotoxemia”^1^,^2^. Gut permeability and endotoxemia seem to play crucial role in the pathogenesis of alcoholic liver disease and potentially in alcoholic damage to other organs. Therefore, the loss of mucosal barrier function and increased gut permeability play a central role in the mechanism of alcoholic tissue injury. Tissues *in vivo* are simultaneously exposed to ethanol and acetaldehyde due to ubiquitous distribution of alcohol dehydrogenase (ADH)^3^, CYP2E1^4^ and catalase^5^,^6^, all known to metabolize ethanol into acetaldehyde. Mounting evidence indicates that ethanol metabolism by ADH into acetaldehyde plays a crucial role in alcoholic tissue injury in many organs^7^,^8^,^9^,^10^,^11^,^12^,^13^. Studies reported so far have evaluated the effect of either ethanol or acetaldehyde on tissue functions, including the epithelial barrier function. In the present study, we addressed the potential cross talk between ethanol and acetaldehyde in disrupting the intestinal epithelial tight junction integrity and barrier function.

Epithelial tight junctions confer the intestinal mucosal barrier function in the intestine. Tight junctions are multi protein complexes consisting of transmembrane proteins such as occludin, tricellulin, claudins and junctional adhesion molecules and adapter proteins such as ZO-1, ZO-2 and ZO-3, which interact with many other proteins including actin-binding proteins^14^,^15^,^16^. Tight junction protein complexes are anchored to the actin cytoskeleton^15^. Protein-protein interactions and the association with the actin cytoskeleton are essential for the assembly and maintenance of tight junction integrity. Many signaling molecules, including protein kinases and proteins phosphatases, interact with the tight junction proteins, and the tight junction integrity is regulated by intracellular signal transduction^17^.

Acetaldehyde has been shown to disrupt the intestinal epithelial tight junctions and increase paracellular permeability to endotoxins^1^,^2^. Acetaldehyde inhibits protein tyrosine phosphatases and increases tyrosine phosphorylation of tight junction and adherens junction proteins^18^,^19^. Acetaldehyde also induces translocation of protein phosphatase PP2A to tight junctions and dephosphorylates tight junction proteins on threonine residues^20^. The role of ethanol metabolism into acetaldehyde in the intestinal permeability was confirmed in rat intestine *ex vivo*^21^ and mouse colon *in vivo*^22^. Therefore, the role of acetaldehyde in disruption of intestinal epithelial tight junction is well established. But, the direct effect of ethanol on epithelial permeability is controversial. Few studies failed to show an effect of ethanol on barrier function at concentrations lower than 2.5%^23^,^24^,^25^,^26^, whereas other studies have recorded a direct effect of ethanol on the barrier function in Caco-2 cell monolayers^27^. The simultaneous exposure of tissues to ethanol and acetaldehyde in alcoholics raises the question whether one influences the effect of other in causing tissue injury.

In the present study, we investigated the synergistic effect of ethanol and acetaldehyde on the tight junction integrity and barrier function in Caco-2 cell monolayers, and delineated the role of calcium influx and mitochondrial oxidative stress in the mechanism involved in the synergistic effect of ethanol and acetaldehyde.

## Results

### Ethanol metabolism plays a role in tight junction disruption and barrier dysfunction

**Figure 1:**
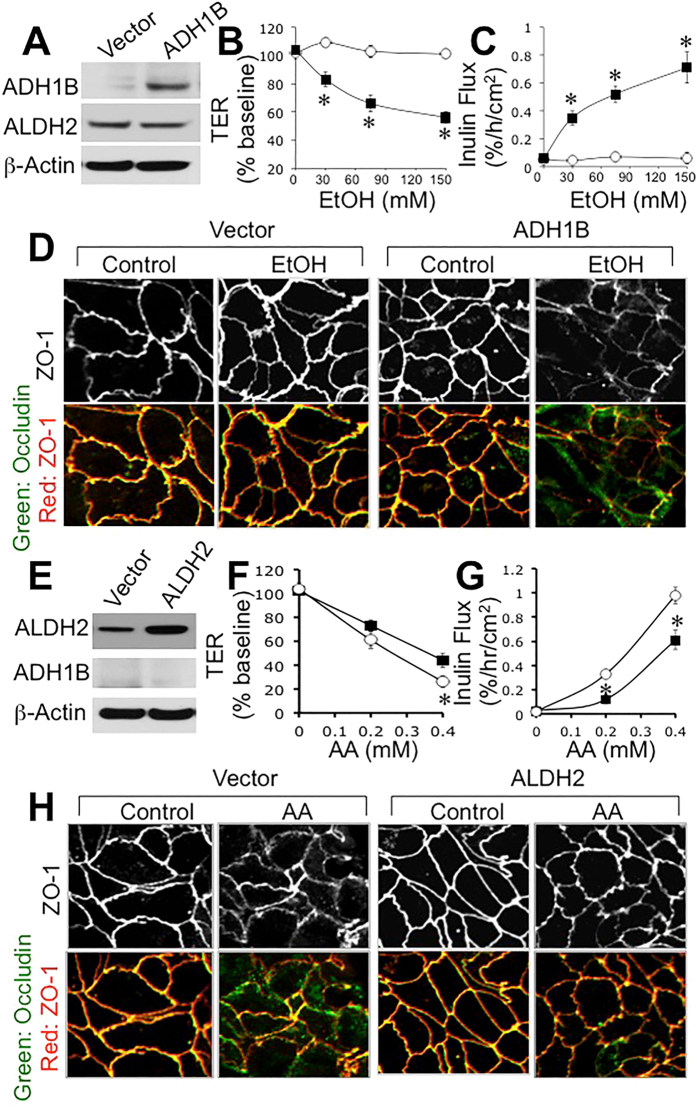
Expression of ADH1B and ALDH2 alters ethanol and acetaldehyde-induced tight junction disruption and barrier dysfunction. Caco-2 cells were transfected with ADH1B (closed symbols) or vector (open symbols). Expression of ADH1B in transiently transfected cells was determined by immunoblot analysis (**A**). Vector and ADH1B-transfected cells in transwell inserts were incubated with varying concentrations of ethanol. At 3-hour incubation, TER (**B**) and unidirectional flux of FITC-inulin (**C**) were measured. Fixed cell monolayers were stained for occludin (green) and ZO-1 (red) by immunofluorescence method (**D**). Values in panels B and C are mean ± SEM (n = 6). Asterisks indicate values that are significantly (p < 0.05) different from corresponding values for control cell monolayers. Caco-2 cells were transfected with ALDH2 or vector. Expression of ALDH2 in transiently transfected cells was determined by immunoblot analysis (**E**). Vector (open symbols) and ALDH2 (closed symbols)-transfected cells in transwell inserts were incubated with varying concentrations of acetaldehyde. At 4-hour incubation, TER (**F**) and unidirectional flux of FITC-inulin (**G**) were measured. Fixed cell monolayers were stained for occludin (green) and ZO-1 (red) by immunofluorescence method (**H**). Values in panels B and C are mean ± SEM (n = 6). Asterisks indicate values that are significantly (p < 0.05) different from corresponding values for control cell monolayers.

Caco-2 cells express high level of aldehyde dehydrogenase (ALDH), but only trace level of ADH is detected^28^. Previous studies showed that acetaldehyde is more effective than ethanol in tight junction disruption in Caco-2 cell monolayer^1^. To confirm the role of ethanol metabolism on tight junction disruption Caco-2 cells were transfected with ADH1B or ALDH2. Immunoblot analysis showed only trace amount of ADH in vector-transfected cell monolayers, whereas ADH1B-transfected cells showed high level of ADH (Fig. 1A). Incubation of vector-transfected cells with ethanol up to 150 mM caused no significant effect on transepithelial electrical resistance (TER) (Fig. 1B) or inulin permeability (Fig. 1C). But, ethanol dose-dependently reduced TER and increased inulin permeability in ADH1B-transfected cell monolayers. Confocal immunofluorescence microscopy showed that ethanol produced no obvious effect on junctional distribution of occludin and ZO-1, but it caused a dramatic redistribution of occludin and ZO-1 from the intercellular junctions into intracellular compartment in ADH1B-transfected cell monolayers (Fig. 1D).

Higher level of ALDH was detected in ALDH2-transfected cells compared to that in vector-transfected cells (Fig. 1E). Incubation with acetaldehyde induced dose-dependent decrease in TER (Fig. 1F) and increase in inulin permeability (Fig. 1G), and these effects were significantly dampened in ALDH2-transfected cell monolayers. Acetaldehyde-induced redistribution of occludin and ZO-1 from the intercellular junctions was also diminished in ALDH2-transfected cell monolayers compared to that in vector-transfected cell monolayers (Fig. 1H).

### Ethanol synergizes acetaldehyde-induced tight disruption and barrier dysfunction

**Figure 2:**
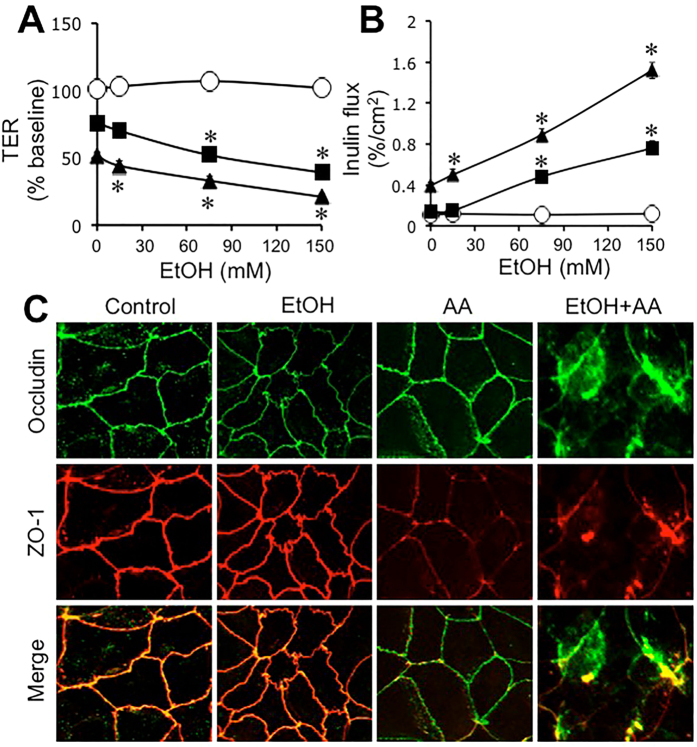
Ethanol and acetaldehyde induce synergistic disruption of tight junctions and barrier dysfunction. Caco-2 cells were incubated with acetaldehyde (○, 0 μM; ■, 100 μM; ▲, 200 μM) in the presence of varying concentrations of ethanol. At 4-hour incubation, TER (**A**) and unidirectional flux of FITC-inulin (**B**) were measured. Fixed cell monolayers were stained for occludin (green) and ZO-1 (red) by immunofluorescence method (**C**). Values are mean ± SEM (n = 6). Asterisks indicate values that are significantly (p < 0.05) different from corresponding values for control cell monolayers (without ethanol).

To determine the influence of ethanol on acetaldehyde effect we evaluated acetaldehyde-induced barrier dysfunction and tight junction disruption in the presence of varying concentrations of ethanol. In the absence of acetaldehyde, ethanol up to 150 mM showed no significant effect on TER (Fig. 2A). In the absence of ethanol, acetaldehyde at 100 μM and 200 μM concentrations reduced TER by 20% and 50%, respectively. These effects of acetaldehyde on TER were dose-dependently elevated by ethanol (Fig. 2A). Similarly, ethanol in the absence of acetaldehyde produced no significant effect on inulin permeability. In the absence of ethanol, acetaldehyde at 100 μM concentration produced no significant effect on inulin permeability, and produced only slight increase in inulin permeability at 200 μM concentration (Fig. 2B). Acetaldehyde effect on inulin permeability at both 100 μM and 200 μM concentrations were dose-dependently elevated by ethanol. Immunofluorescence images show that ethanol in the absence of acetaldehyde caused no obvious effect on junctional distribution of occludin and ZO-1, but acetaldehyde (200 μM) in the absence of ethanol showed a partial depletion of junctional organization of occludin and ZO-1 (Fig. 2C). Acetaldehyde in the presence of ethanol caused a dramatic redistribution of occludin and ZO-1 from the intercellular junctions into intracellular compartments (Fig. 2C).

**Figure 3:**
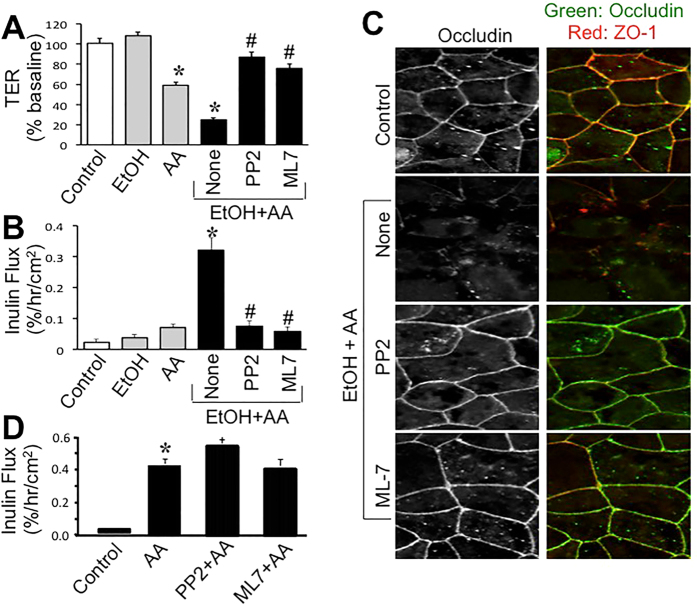
Src kinase and MLCK activities mediate ethanol and acetaldehyde-induced tight junction disruption and barrier dysfunction. (**A–C**) Caco-2 cells were preincubated with PP2 (10 μM) or ML-7 (1 μM) 30 min prior to incubation with acetaldehyde (200 μM) in the presence of 75 mM ethanol. At 4-hour incubation, TER (**A**) and unidirectional flux of FITC-inulin (**B**) were measured. Fixed cell monolayers were stained for occludin (green) and ZO-1 (red) by immunofluorescence method (**C**). Values are mean ± SEM (n = 6). Asterisks indicate values that are significantly different from corresponding values for control cell monolayers (without ethanol). (**D**) Cell monolayers were preincubated with PP2 (10 μM) or ML-7 (1 μM) 30 min prior to incubation with acetaldehyde (400 μM). Inulin permeability was measured after 4-hour incubation. Values are mean ± SEM (n = 6). Asterisk indicates the value that is significantly (p < 0.05) different from control value.

To determine the signaling mechanism associated with the synergistic effect of ethanol and acetaldehyde we evaluated the effect of Src kinase inhibitor (PP2) and myosin light chain kinase (MLCK) inhibitor (ML-7). Both PP2 and ML-7 significantly blocked ethanol and acetaldehyde-induced decrease in TER (Fig. 3A) and increase in inulin permeability (Fig. 3B). Immunofluorescence images show that both PP2 and ML-7 blocked ethanol and acetaldehyde-induced redistribution of occludin and ZO-1 from the intercellular junctions (Fig. 3C). Previous studies showed that acetaldehyde at higher concentration (400 μM) causes barrier dysfunction in the absence of ethanol. We evaluated the effect of PP2 and ML-7 on the effect of higher dose of acetaldehyde. Acetaldehyde increased inulin permeability by several folds, but PP2 or ML-7 failed to block this effect of acetaldehyde (Fig. 3D).

### Ethanol and acetaldehyde synergistically elevate intracellular calcium ([Ca^2+^]_i_) leading to tight junction disruption

**Figure 4:**
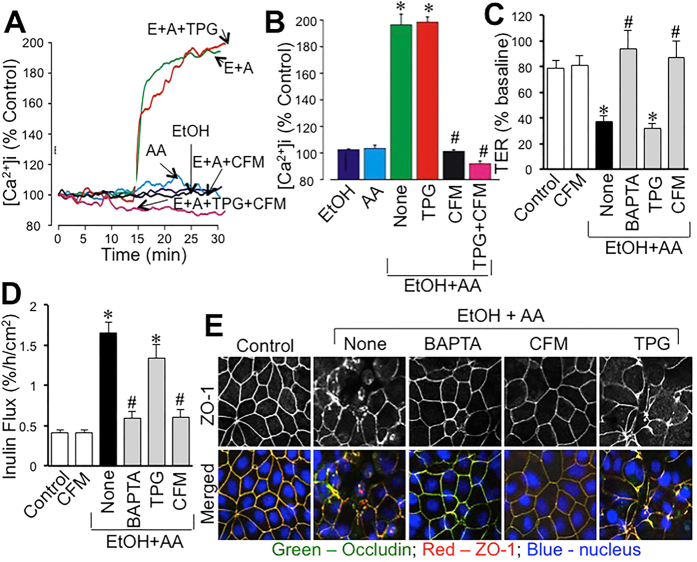
Synergistic elevation of intracellular Ca^2+^ by ethanol and acetaldehyde mediates tight junction disruption. (**A,B**) Fura-2 loaded Caco-2 cell monolayers were incubated with 75 mM ethanol (EtOH), 200 μM acetaldehyde (AA) or acetaldehyde (200 μM) added 10 min after 75 mM ethanol (E+A) in the absence or presence of thapsigargin (TPG) or Ca^2+^-free medium (CFM). Real-time change in [Ca^2+^]_i_ was measured and quantitated. Values are mean ± SEM (*n* = 10). Asterisks indicate the values that are significantly (p < 0.05) different from corresponding basal values; hash tags indicate values that are significantly (p < 0.05) different from value for E+A group. (**C–E**) Caco-2 cell monolayers were incubated with acetaldehyde (200 μM) added 10 min after 75 mM ethanol (EtOH+AA) in the absence (●) or presence of BAPTA-AM, TG, or CFM. Control cell monolayers received no treatments. TER (**C**) and inulin permeability (**D**) were measured after 4-hour incubation. Values are mean ± SEM (*n* = 8). Asterisks indicate the values that are significantly different (p < 0.05) from corresponding control values; hash tags indicate values that are significantly (p < 0.05) different from value for “None” group (●). Cell monolayers were fixed and stained for occludin and ZO-1 (**E**).

The effect of ethanol and acetaldehyde on [Ca^2+^]_i_ was evaluated by live cell fluorescence imaging. Application of ethanol alone up to 1% concentration produced no significant effect on [Ca^2+^]_i_ at least up to 30 min. Similarly, acetaldehyde alone up to 400 μM caused no significant change in [Ca^2+^]_i_ (Fig. 4A). Interestingly, application of 200 μM acetaldehyde 10 min after ethanol (75 mM) treatment induced a rapid increase in [Ca^2+^]_i_, which sustained for at least 15 min. Incubation of cells in Ca^2+^-free medium abolished ethanol and acetaldehyde-induced rise in [Ca^2+^]_i_ (Fig. 4A and B). Pretreatment of cells with thapsigargin (TPG) did not alter ethanol and acetaldehyde-induced elevation of [Ca^2+^]_i_. Ethanol and acetaldehyde-induced decrease in TER (Fig. 4C) and increase in inulin permeability (Fig. 4D) were blocked by BAPTA and Ca^2+^-free medium, but not by thapsigargin (TPG). Ethanol and acetaldehyde-induced redistribution of occludin and ZO-1 from the intercellular junctions was also blocked by BAPTA and Ca^2+^-free medium (Fig. 4E).

### Ca^2+^ channels are involved in ethanol and acetaldehyde-induced tight junction disruption

**Figure 5:**
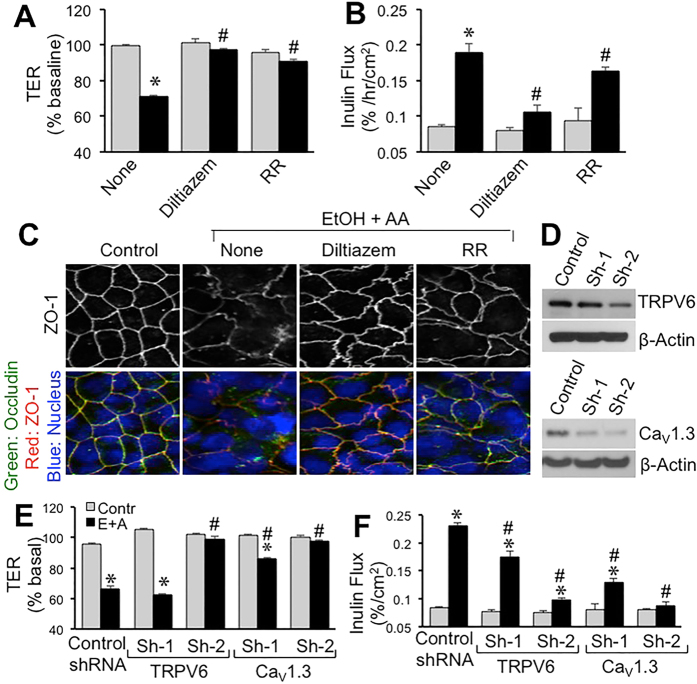
Ca^2+^ channels play role in ethanol and acetaldehyde-induced disruption of tight junctions and barrier dysfunction. (**A–C**) Caco-2 cells were preincubated with diltiazem (30 μM) or ruthenium red (RR; 30 μM) 30 min prior to incubation with acetaldehyde (200 μM) in the presence of 75 mM ethanol (closed bars; EtOH+AA) or without acetaldehyde or ethanol treatment (gray bars). At 4-hour incubation, TER (**A**) and unidirectional flux of FITC-inulin (**B**) were measured. Fixed cell monolayers were stained for occludin (green) and ZO-1 (red) by immunofluorescence method (**C**). Values are mean ± SEM (n = 6). Asterisks indicate values that are significantly different from corresponding values for control cell monolayers (gray bars). Hash tags indicate values that are significantly different from corresponding values for “None” group. (**D–F**) Caco-2 cells were transfected with either of two different shRNA (Sh1 and Sh2) each for Ca_V_1.3 and TRPV6 or control shRNA and down regulation of specific channels was confirmed by immunoblot analysis for corresponding channels (**D**). Transfected cell monolayers were treated with 100 mM ethanol followed by 200 μM acetaldehyde. TER (**E**) and inulin permeability (**F**) were measured. Values are mean ± SEM (n = 6). Asterisks indicate the values that are significantly different from corresponding control values (gray bars). Hash tags indicate values that are significantly different from corresponding values for “Control shRNA” group.

To determine the role of Ca^2+^ channels we evaluated the effect of diltiazem and ruthenium red on ethanol and acetaldehyde effects. Both diltiazem and ruthenium red significantly attenuated ethanol and acetaldehyde-induced decrease in TER (Fig. 5A) and increase in inulin permeability (Fig. 5B). Diltiazem was more effective than ruthenium red in blocking the effects of ethanol and acetaldehyde. Ethanol and acetaldehyde-induced redistribution of occludin and ZO-1 from the intercellular junctions was also blocked by diltiazem (Fig. 5C). Ruthenium red caused only a partial prevention of ethanol and acetaldehyde-induced redistribution of occludin and ZO-1.

Calcium channels, Ca_V_1.3 and TRPV6, were knocked down by specific shRNAs (Fig. 5D). Ethanol and acetaldehyde-induced decrease in TER (Fig. 5E) and increase in inulin permeability (Fig. 5F) were significantly low in Ca_V_1.3 and TRPV6-deficient cell monolayers compared to those in control RNA-transfected cell monolayers.

### Ethanol and acetaldehyde synergistically increase reactive oxygen species (ROS)

**Figure 6:**
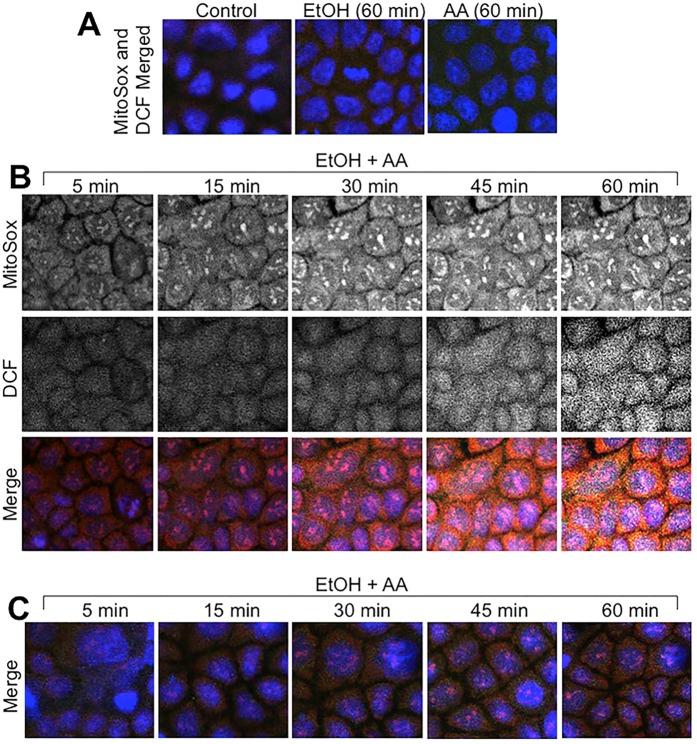
Ethanol and acetaldehyde induces a synergistic production of ROS by a Ca^2+^-dependent mechanism. MitoSOX^TM^ and H_2_DCF-DA-loaded Caco-2 cell monolayers were incubated with 75 mM ethanol (EtOH) or 200 μM acetaldehyde (AA) (**A**) or acetaldehyde (200 μM) added 10 min after 75 mM ethanol (EtOH+AA) in the absence (**B)** or presence (**C**) of Ca^2+^-free medium. Real-time change in [Ca^2+^]_i_ was imaged at varying times.

Mitochondrial Ca^2+^ overload is known to induce oxidative stress^29^. Sustained elevation of [Ca^2+^]_i_ raised the question whether ethanol and acetaldehyde induce ROS production. Therefore, we examined the effect of ethanol and acetaldehyde on ROS production in Caco-2 cells by live cell fluorescence imaging. Ethanol, in the absence of acetaldehyde or acetaldehyde in the absence of ethanol, failed to increase MitoSOX^TM^ or dichlorofluorescein (DCF)-sensitive fluorescence (Fig. 6A). But, acetaldehyde and ethanol together rapidly increased MitoSOX^TM^ and DCF-sensitive fluorescence reaching a maximum by 45 min (Fig. 6B). MitoSOX^TM^ is specific for mitochondrial superoxide and DCF is relatively non-specific. Incubation of cells with Ca^2+^-free medium blocked ethanol and acetaldehyde-induced elevation of MitoSOX^TM^ and DCF-sensitive fluorescence (Fig. 6C).

**Figure 7:**
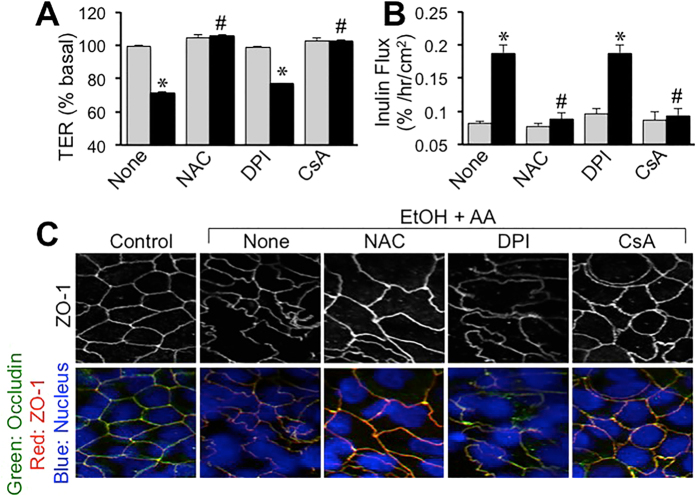
Oxidative stress mediates ethanol and acetaldehyde-induced tight junction disruption and barrier dysfunction. Caco-2 cells were preincubated with NAC (10 mM), DPI (10 μM) or CsA (5 μM) 30 min prior to incubation with acetaldehyde (200 μM) in the presence of 75 mM ethanol (closed bars; EtOH+AA) or without acetaldehyde or ethanol treatment (gray bars). At 4-hour incubation, TER (**A**) and unidirectional flux of FITC-inulin (**B**) were measured. Fixed cell monolayers were stained for occludin (green) and ZO-1 (red) by immunofluorescence method (**C**). Values are mean ± SEM (n = 6). Asterisks indicate values that are significantly different from corresponding values for control cell monolayers (gray bars). Hash tags indicate values that are significantly different from corresponding values for “None” group.

To determine the role of oxidative stress in ethanol and acetaldehyde-induced tight junction disruption and barrier dysfunction we evaluated the effect of antioxidants. Pretreatment of cell monolayers with N-acetyl L-cysteine (NAC) blocked ethanol and acetaldehyde-induced decrease in TER (Fig. 7A) and increase in inulin permeability (Fig. 7B). NAC also attenuated ethanol and acetaldehyde-induced redistribution of occludin and ZO-1 from the intercellular junctions (Fig. 7C). Pretreatment with diphenyleneiodonium (DPI) failed to affect ethanol and acetaldehyde-induced changes in TER, inulin flux or redistribution of occludin and ZO-1 from the intercellular junctions. On the other hand, cyclosporine A (CsA) treatment blocked ethanol and acetaldehyde-induced decrease in TER (Fig. 7A), inulin permeability (Fig. 7B) and redistribution of occludin and ZO-1 from the junctions (Fig. 7C).

## Discussion

Exposure of tissues simultaneously to ethanol and acetaldehyde is a realistic scenario in alcoholics *in vivo*. However, the information we have today were derived from studies evaluating the effect of ethanol^30^ or acetaldehyde^1^,^21^,^23^,^25^,^26^,^30^ alone. The present study shows that ethanol and acetaldehyde exert a synergistic effect on the intestinal epithelial barrier function in Caco-2 cell monolayers. Furthermore, this study presents evidence to the potential mechanisms involved in the synergistic effect of ethanol and acetaldehyde on epithelial barrier function.

Mounting evidence indicates that ethanol metabolism and acetaldehyde production play crucial role in ethanol-induced tight junction disruption and barrier dysfunction in the intestinal epithelium^1^. Few recent studies indicated that ethanol directly increases intestinal epithelial permeability in Caco-2 cell monolayers^30^, but our studies have consistently showed a significant resistance of Caco-2 cell monolayers to ethanol-induced barrier dysfunction^1^. Although catalase and CYP2E1 are known to metabolize ethanol into acetaldehyde, ADH is the primary mechanism of ethanol metabolism^3^. Following the liver, gut is the organ with high expression of ADH^3^. Caco-2 cells however express only trace amounts of alcohol dehydrogenase (ADH)^28^. Accordingly, previous studies showed that ethanol disrupts barrier function of Caco-2 cell monolayers only at high concentrations^23^,^25^,^26^, whereas its metabolic product acetaldehyde was highly effective in barrier disruption^1^. Immunoblot analysis in the present study confirms lack of ADH in Caco-2 cells. The present study shows that ethanol up to 150 mM concentration has no significant effect on the barrier function in Caco-2 cell monolayers. But, over expression of ADH1B increased sensitivity to ethanol-induced tight junction disruption and barrier dysfunction in Caco-2 cell monolayers, indicating that ethanol metabolism and generation of acetaldehyde is likely required for epithelial tight junction disruption. This is further confirmed by our observation that over expression of ALDH2 dampened the acetaldehyde effect on barrier function and tight junction integrity in Caco-2 cell monolayers. Variable sensitivity of Caco-2 cells for ethanol-induced tight junction disruption reported by different laboratories is possibly due to the differences in the sub clones of Caco-2 used, the passage numbers and the age of cell monolayers with different levels of ADH expression.

Due to ethanol metabolism in cells, the tissues *in vivo* are simultaneously exposed to ethanol and acetaldehyde. But, ethanol or acetaldehyde alone has been used to evaluate their effects on the intestinal epithelial barrier function in *in vitro* studies. In the present study, we show that ethanol up to 150 mM exerts no influence on barrier function in Caco-2 cell monolayers, but it dose-dependently increases the effect of acetaldehyde on barrier function and tight junction integrity. Data in Fig. 1 show that acetaldehyde is clearly required for tight junction disruption and barrier dysfunction. But, data in this figure does not rule out an influence of ethanol on the acetaldehyde-induced tight junction disruption. Data presented in Fig. 2 indicate that ethanol enhances the effect of acetaldehyde at low concentrations, although ethanol by itself shows no effect on barrier function. Acetaldehyde alone at 100 μM concentration showed no effect on inulin permeability, whereas in the presence of ethanol it significantly increased inulin permeability; this effect of ethanol was dose-dependent. These data indicate that ethanol and low concentration of acetaldehyde synergistically disrupt intestinal epithelial tight junctions and cause barrier dysfunction. It is likely that a similar synergy exists *in vivo*; acetaldehyde and ethanol are likely to cause tissue injury at concentrations lower than those reported *in vitro* studies. We evaluated the potential synergism between acetate (the product of ALDH activity) and ethanol on barrier function, but found no significant effect on barrier function. Similarly, pretreatment of cell monolayers with *E. coli* lipopolysaccharide (LPS) also had no influence on ethanol and acetaldehyde-induced barrier dysfunction.

Prevention of the ethanol and acetaldehyde-induced changes in TER, inulin permeability and redistribution of occludin and ZO-1 by PP2 indicated that Src kinase activity is involved in the synergistic effect of ethanol and acetaldehyde on barrier function and tight junction integrity. Src kinases, particularly c-Src, have been previously shown to mediate in tight junction disruption by hydrogen peroxide^31^, osmotic stress^32^ and dextran sulfate sodium^33^. Tyrosine phosphorylation of tight junction proteins has been shown to prevent protein-protein interactions leading to disruption of tight junction^34^,^35^. Requirement of Src kinase activity in the present study suggests that tyrosine kinases such as c-Src and tyrosine phosphorylation of tight junction proteins may be involved in the synergistic disruption of tight junction and barrier function by ethanol and acetaldehyde. Interestingly, Src kinase activity is not involved in barrier dysfunction and tight junction disruption caused by a higher concentration of acetaldehyde in the absence of ethanol. Therefore, distinct mechanisms are likely to be involved in tight junction disruption by acetaldehyde alone and the synergistic effect of ethanol and acetaldehyde. Previous studies showed that acetaldehyde increases tyrosine phosphorylation of tight junction proteins, but it was mediated by inhibition of protein tyrosine phosphatases^18^,^19^.

The synergistic effect of ethanol and acetaldehyde on TER, inulin permeability and redistribution of occludin and ZO-1 was blocked by ML-7 indicating the potential role of MLCK in ethanol and acetaldehyde-induced tight junction disruption and barrier dysfunction. MLCK activity was shown to mediate the disruption of intestinal epithelial tight junction and barrier dysfunction by TNFα^36^,^37^,^38^ and hydrogen peroxide^39^. The original study by Ma and coworkers showed that tight junction disruption by higher concentration of ethanol in Caco-2 cell monolayers was mediated by MLCK^23^.

A recent study showed a potential role of [Ca^2+^]_i_ in ethanol-induced tight junction disruption^27^. MLCK activity is known to be regulated by [Ca^2+^]_i_. Therefore, we examined the effect of ethanol and acetaldehyde on [Ca^2+^]_i_. Interestingly, acetaldehyde applied following ethanol treatment rapidly increased [Ca^2+^]_i_ and sustained for at least 15 min. This observation indicates that ethanol and acetaldehyde synergistically elevates [Ca^2+^]_i_. To determine the source of elevated [Ca^2+^]_i_ we evaluated the effect of Ca^2+^-free medium and TPG. A complete attenuation of ethanol and acetaldehyde-mediated elevation of [Ca^2+^]_i_ by Ca^2+^-free medium indicates that influx of extracellular Ca^2+^ is involved in ethanol and acetaldehyde-induced elevation of [Ca^2+^]_i_. The lack of an effect of TPG indicates that Ca^2+^ release from endoplasmic reticulum is not involved in the effect of ethanol and acetaldehyde on [Ca^2+^]_i_ on tight junction integrity. Elevation of [Ca^2+^]_i_ raised the question whether [Ca^2+^]_i_ is involved in the synergistic disruption of tight junction by ethanol and acetaldehyde. Depletion of [Ca^2+^]_i_ by BAPTA or incubation in Ca^2+^-free medium abrogated ethanol and acetaldehyde-induced barrier dysfunction and tight junction disruption. These results demonstrate that elevation of [Ca^2+^]_i_ plays a key role in the synergistic effect of ethanol and acetaldehyde on tight junction integrity and barrier function.

The role of Ca^2+^ influx in ethanol and acetaldehyde-induced elevation of [Ca^2+^]_i_ raised the question whether Ca^2+^ channels such as Ca_V_1.3 and TRPV are involved in ethanol and acetaldehyde-induced barrier dysfunction. The results of our present study show that diltiazem, the voltage-gated channel blocker, attenuates the synergistic effect of ethanol and acetaldehyde on TER, inulin permeability and redistribution of occludin and ZO-1. This observation indicates that voltage-gated Ca^2+^ channels are involved in the disruption of tight junction and barrier dysfunction by ethanol and acetaldehyde. Ruthenium red, a selective blocker of TRPV channels and ryanodine receptors produced a partial block of the synergistic effect of ethanol and acetaldehyde on barrier function and tight junction integrity. The ruthenium red effects suggest that TRPV type of Ca^2+^ channels may also be involved in ethanol and acetaldehyde-induced tight junction disruption and barrier dysfunction. Ca_V_1.3 and TRPV6 are two of the voltage-gated Ca^2+^ channels located in the apical membrane of intestinal epithelium. Specific knockdown of these channels by shRNA transfection in Caco-2 cells showed that both these channels are required for ethanol and acetaldehyde-induced barrier dysfunction.

To further elaborate the mechanism of synergistic effects of ethanol and acetaldehyde on tight junction and barrier function, we investigated the potential role of oxidative stress in ethanol and acetaldehyde effects. Previous studies have demonstrated that oxidative stress disrupts intestinal epithelial tight junction by activating Src kinase^31^. Elevation of [Ca^2+^]_i_ is known to induce mitochondrial oxidative stress^40^. The present study shows that ethanol or acetaldehyde alone does not induce ROS production in Caco-2 cells. However, when the acetaldehyde was applied after ethanol treatment there was a rapid and time-dependent increase in the levels of both MitoSox-sensitive and DCF-sensitive ROS. A maximum level of ROS produced was achieved by 30–45 min. MitoSox-sensitive ROS production is known to represent superoxide^41^, whereas DCF-sensitive ROS represent most types of ROS, including hydrogen peroxide and hydroxyl radicals^42^. The results of present study indicate that ethanol and acetaldehyde synergistically induce ROS production in Caco-2 cells, which is parallel to synergistic effect of ethanol and acetaldehyde on barrier function and [Ca^2+^]_i_. Prevention of ethanol and acetaldehyde-induced ROS by Ca^2+^-free medium indicates that the elevation of [Ca^2+^]_i_ plays a crucial role in ethanol and acetaldehyde-induced ROS production. Ethanol and acetaldehyde-induced ROS production was also blocked by CsA. CsA is an inhibitor of mitochondrial membrane permeability transition (MMPT)^43^, suggesting that MMPT is likely involved in ROS production. We measured the levels of NAD and NADH in cell monolayers treated with ethanol and acetaldehyde. The data show that ethanol and acetaldehyde treatment did increase NADH and NADH/NAD ratio, and the effect was maintained to be higher in ADH1B-transfected cells (Fig. S2).

Synergistic production of ROS raised the question whether oxidative stress played a role in ethanol and acetaldehyde-induced tight junction disruption and barrier dysfunction. Pretreatment of cell monolayers with NAC blocked ethanol and acetaldehyde-induced changes in TER, inulin permeability and redistribution of occludin and ZO-1, indicating that oxidative stress plays a role in ethanol and acetaldehyde-induced tight junction disruption and barrier dysfunction. The lack of an effect of DPI indicates that NADPH oxidase does not play a role in ethanol and acetaldehyde-induced oxidative stress and tight junction disruption. Interestingly, CsA blocked ethanol and acetaldehyde-induced changes in TER, inulin permeability and redistribution of occludin and ZO-1, indicating that MMPT and mitochondrial oxidative stress are involved in the synergistic disruption of tight junction and barrier function by ethanol and acetaldehyde in the intestinal epithelium.

**Figure 8:**
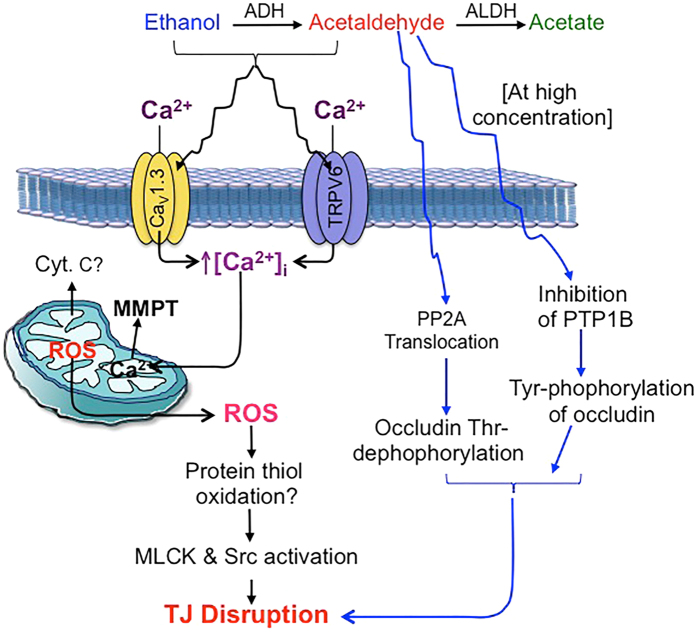
The potential mechanism involved in the synergistic disruption of tight junctions by ethanol and acetaldehyde. At low acetaldehyde concentration (cascade with black arrows on the left), the ethanol and acetaldehyde synergistically elevate [Ca^2+^]_i_ in the intestinal epithelium potentially by activating Ca^2+^ channels. Elevated plasma calcium induces oxidative stress likely by causing mitochondrial membrane permeability transition. Oxidative stress-induced protein-thiol oxidation inhibits PTP1B and activates c-Src and MLCK resulting in disruption of epithelial tight junctions. At high acetaldehyde concentration (cascade with blue arrows on the right), it disrupts tight junctions in the absence of ethanol by inhibition of PTP1B leading to Tyr-phosphorylation of occludin and other tight junction proteins, and by translocation of PP2A to tight junction leading to dephosphorylation of occludin on Thr residues.

In summary, this study provides evidence that ethanol and acetaldehyde synergistically disrupt intestinal epithelial tight junction and induce barrier dysfunction. As illustrated in the figure data indicates the mechanism of synergistic disruption of tight junctions by ethanol and acetaldehyde involves elevation of [Ca^2+^]_i_, mitochondrial oxidative stress and activities of Src kinase and MLCK (Fig. 8). This study reveals a new dimension to the role of ethanol metabolism and acetaldehyde production in alcoholic gut permeability and endotoxemia.

## Methods

### Chemicals

Cell culture supplies, transfection reagents, Fura-2AM and pluronic acid were procured from Cellgro^®^ (Manassas, VA) or Invitrogen (Carlsbad, CA). Transwells were purchased from Costar (Cambridge, MA). TPG, ML-7, CsA and PP2 were from EMD Chemicals (San Diego, CA). Other chemicals were purchased from either Sigma Aldrich (St. Louis, MO) or Thermo Fisher Scientific (Tustin, CA). MitoSOX^TM^ and H_2_DCFDA were procured from ThermoFisher:Life Technologies (Grand Island, NY).

### Antibodies

Anti-ZO-1 and anti-occludin antibodies were purchased from Invitrogen (Carlsbad, CA). Anti ADH1B and anti-ALDH2 antibodies were procured from Origene Technologies (Atlanta, GA). Anti-TRPV6 antibody was purchased from Santa Cruz Biotechnology (Dallas, TX) and anti-Ca_V_1.3 antibody was procured from abcam (Cambridge, MA). HRP-conjugated anti-mouse IgG, HRP-conjugated anti-rabbit IgG and anti-β-actin antibodies were obtained from Sigma Aldrich. AlexaFlour-488-conjugated anti-mouse IgG and Cy3-conjugated anti-rabbit IgG were purchased from Molecular Probes (Eugene, OR).

### Expression constructs

ADH1B (SKU: SC335414) and ALDH2 (SKU: MC217268) cDNA in pCMV vector were purchased from Origene (Rockville, MD). A vector-based short hairpin RNA (shRNA) method was used to silence Ca_V_1.3 and TRPV6 gene expression in Caco-2 cells. Two targeting sequences each were chosen against the nucleotide sequence of human *Ca*_*V*_*1.3* (Gene ID: 776 CACNAID; NM_000720.2) and human *TRPV6* (Gene ID: ECAC2; NM_018646) using Dharmacon siDesigner software: Target 1, CCGAATAGCTCCAAGCAAA (sequence position 290–308) and Target 2, GGAAGACCCAGAGATACA (sequence position 5697–5715) for *Ca*_*V*_*1.3,* and Target 1, GACAAAGACTCAGTGGAA (sequence position 2207–2224) and Target 2, GAAACAGCGCTACACATA (sequence position 467–484) for *TRPV6*. To construct shRNA vectors, two pairs of oligonucleotides containing the antisense sequence, hairpin loop region (TTGATATCCG), and sense sequence with cohesive BamHI and HindIII sites were synthesized (Integrated DNA Technologies Inc., Coralville, IA).

### Cell culture and transfection

Caco-2_bbe2_ cells (ATCC, Rockville, MD) were grown under standard cell culture conditions as Previously described^44^. Experiments were conducted using cells grown in transwell inserts of varying diameters (6.5 or 12 mm) for 3–4 or 15–17 days.

Cells grown in 6-well costar plates for 24 h showing ≅75% confluence were transfected, using 1 ml antibiotics-free DMEM containing 10% FBS, 1 μg DNA plasmid (Empty vector, control or the plasmid DNA carrying the tyrosine mutations), 1 μl Plus reagent, and 3 μl Lipofectamine-R for each well. After 20 hours, the cell monolayers were trypsinized and seeded on to transwell inserts (6.5 mm diameter). Cell monolayers on day 3 or 4 post seeding were used to evaluate the effect of ethanol and acetaldehyde on barrier function.

### Cell treatments

Cell monolayers in transwell inserts were incubated with ethanol (0–150 mM), acetaldehyde (100–400 μM) or acetaldehyde (100 or 200 μM) applied 10 min after ethanol (75 mM) treatment as described before^18^. In some experiments cell monolayers were pretreated with PP2 (10 μM), ML-7 (1 μM), CsA (20 μM), NAC (10 mM), DPI (1 μM) or Ca^2+^-free medium 50 min prior to ethanol treatment. For Ca^2+^ depletion, Ca^2+^ free medium was prepared in DMEM lacking Ca^2+^. Depletion of [Ca^2+^]_i_ was achieved by incubation of cells with 10 μM BAPTA-AM or 1 μM TPG for 30 min.

### Epithelial barrier function

TER was measured using a Millicell-ERS Electrical Resistance System and macromolecular permeability evaluated by measuring unidirectional flux of FITC-inulin as previously described^45^. The basal TER values for Caco-2 cell monolayers in these experiments were 400–500 Ohms/cm^2^.

### Fluorescence microscopy

Caco-2 cell monolayers with 0.2% Triton X-100^TM^, sections were blocked and stained for different proteins as described before^45^. The fluorescence was examined by using a Zeiss LSM 510/710 laser scanning confocal microscope and 20× objective lens. Images from x-y (1 μm) sections were collected using LSM Pascal or Zen software. Images from sections were stacked using ImageJ (NIH) and processed by Adobe Photoshop (Adobe Systems, San Jose, CA).

### Measurement of [Ca^2+^]_i_

[Ca^2+^]_i_ was measured as previously described^32^. Briefly, serum-starved Caco-2 cell monolayers on glass-bottom microwell dishes (MatTek; Ashland, MA) were incubated with Fura-2AM (10 μM) in 0.5% pluronic acid for 30 min. Fura-2-loaded cells were alternately excited at 340 or 380 nm using a PC-driven hyper-switch (Ionoptix; MA, USA). Ratios were collected every second at 510 nm using a Dage MTI iCCD camera and Ionwizard software (Ionoptix). [Ca^2+^]_i_ was calculated using the following equation: [Ca^2+^]_i_ = K_d_ (R−R_min_) (S_f2_)/(R_max_−R) (S_b2_), where R is the 340/380 nm ratio, R_min_ and R_max_ are the minimum and maximum ratios determined in Ca^2+^-free and saturated Ca^2+^ solutions, respectively, S_f2_/S_b2_ is the Ca^2+^ free/Ca^2+^-replete ratio of emissions at 380 nm excitation, and K_d_ is the dissociation constant for Fura-2. R_min_, R_max_, S_f2_, and S_b2_ were determined by increasing the Ca^2+^ permeability of Caco-2 cells with ionomycin (10 μM), and perfusing cells with a high-Ca^2+^ (10 mM) or Ca^2+^-free (10 mM EGTA) solution. The *in situ* apparent dissociation constant (K_d_) for Fura-2 used in this study was 224 nM. Eight to ten cells in each monolayer were analyzed simultaneously, and the experiments were repeated in 3–5 monolayers.

### Detection of ROS production

Caco-2 monolayers were grown on 60 mm dishes to full confluence. Cell monolayers washed with DMEM without phenol red and were incubated with Hoechst 33342 (1:10000) and 10 μM CM-H_2_DCFDA for 40 min at 37 °C in the CO_2_ incubator. Following a wash, cells were incubated with 5 mM of MitoSox for 10 min at 37 °C. Cell monolayers were then exposed to osmotic stress, DSS or cyclic stretch for varying times and fluorescence images were captured by confocal microscope. To determine mitochondrial association of ROS cell monolayers were preincubated with 200 nM Mitotracker green for 30 min followed by incubation with 5 mM Mitosox for 10 min at 37 °C and the images captured 30 min.

### Immunoblot analysis

Proteins in cell extracts were separated by sodium dodecyl sulfate-polyacrylamide gel (7%) electrophoresis and transferred to polyvinylidene fluoride membranes. Membranes were immunoblotted for different proteins as described before^45^.

### NAD/NADH assay

NAD and NADH in cell extracts were measured using Amplite^TM^ Fluorimetric NAD/NADH ratio assay kit (AAT Bioquest, Inc., Sunnyvale, CA). Cells were extracted using the NAD/NADH lysis buffer and the supernatant was used for assay. NAD and NADH levels were measured according to the procedure provided by the vendor. Protein in extract was measured by BCA method, and NAD/NADH values are calculated as nmole per mg protein.

### Statistical analyses

All data are expressed as Mean ± SEM. The differences among multiple groups were first analyzed by ANOVA. When a statistical significance was detected, Tukey’s *t* test was used to determine the statistical significance between multiple testing groups and the corresponding control. Statistical significance was established at 95%.

## Additional Information

**How to cite this article:** Samak, G. *et al*. Calcium Channels and Oxidative Stress Mediate a Synergistic Disruption of Tight Junctions by Ethanol and Acetaldehyde in Caco-2 Cell Monolayers. *Sci. Rep.* **6**, 38899; doi: 10.1038/srep38899 (2016).

**Publisher's note:** Springer Nature remains neutral with regard to jurisdictional claims in published maps and institutional affiliations.

## Supplementary Material

Supplementary Information
