# Supplementary material for: Calcium Channels and Oxidative Stress Mediate a Synergistic Disruption of Tight Junctions by Ethanol and Acetaldehyde in Caco-2 Cell Monolayers
Source: Sci Rep. 2016 Dec 13;6:38899. doi: 10.1038/srep38899 (PMC5153649; doi:10.1038/srep38899)
Supplement: Supplementary Information [file srep38899-s1.pdf]

Supplemental Information
SREP-16-28166-Revised

Calcium Channels and Oxidative Stress Mediate a Synergistic Disruption of Tight Junctions by Ethanol and Acetaldehyde in Caco-2 Cell Monolayers

Geetha Samak*, Ruchika Gangwar*, Avtar S. Meena, Roshan G. Rao, Pradeep K. Shukla, Bhargavi Manda, Damodaran Narayanan, Jonathan H. Jaggar, RadhaKrishna Rao¶

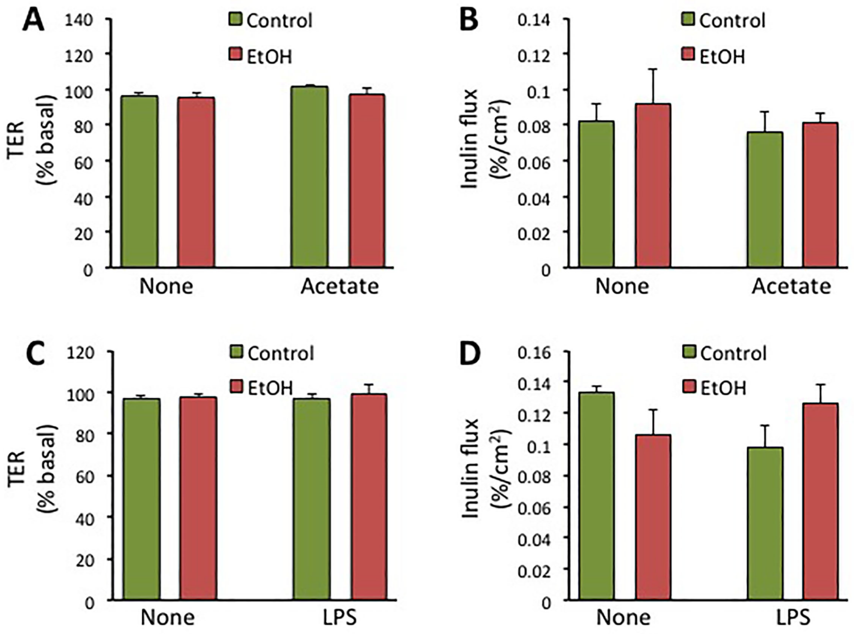

Figure S1: Ethanol fails to synergize with acetate or LPS in disruption of barrier function. A & B: Caco-2 cells were incubated with 1 mM acetate 10 min after ethanol (EtOH) administration (75 mM). At 3-hour incubation, TER (A) and unidirectional flux of FITC-inulin (B) were measured. Values are mean ± SEM (n = 4). **C & D:** Caco-2 cells were incubated with LPS (1 nM) for 16 hours followed by incubation with EtOH (75 mM). At 3-hour incubation with EtOH, TER (C) and unidirectional flux of FITC-inulin (D) were measured. Values are mean ± SEM (n = 4).

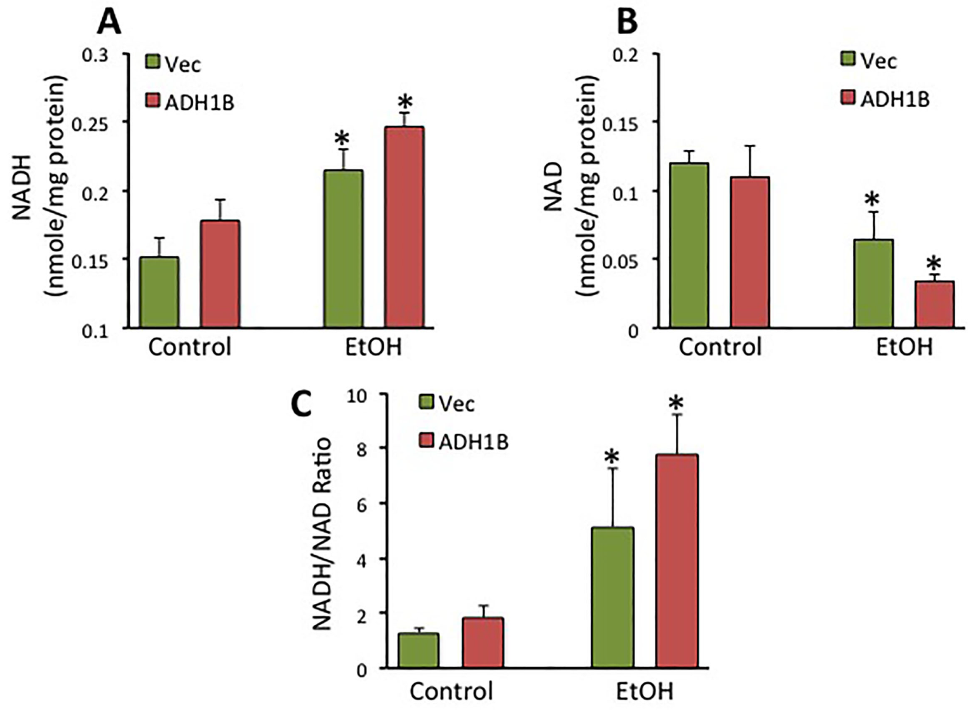

Figure S2: Effect of ethanol and acetaldehyde on NADH/NAD ratio. Caco-2 cells were transfected with ADH1B (closed symbols) or vector (open symbols). Vector and ADH1B-transfected cells were incubated with or without acetaldehyde (200 μM) in the presence of 75 mM ethanol. At 3-hour incubation, cell extracts were analyzed for NADH (A) and NAD (B), and NADH to NAD ratio was calculated (C). Values are mean ± SEM (n = 4). Asterisks indicate the values that are significantly ($p<0.05$) different from corresponding values for control cells.
